# 3-(2*H*-Benzotriazol-2-yl)-2-hydr­oxy-5-methyl­benzaldehyde

**DOI:** 10.1107/S1600536810007233

**Published:** 2010-03-03

**Authors:** Chen-Yu Li, Chen-Yen Tsai, Chia-Her Lin, Bao-Tsan Ko

**Affiliations:** a Department of Chemistry, Chung Yuan Christian University, Chung-Li 32023, Taiwan

## Abstract

In the title compound, C_14_H_11_N_3_O_2_, the dihedral angle between the mean planes of the benzotriazole ring system and the benzene ring of the salicylaldehyde group is 2.4 (2)°. There is an intra­molecular O—H⋯N hydrogen bond which may influence the mol­ecular conformation.

## Related literature

For the application of *N*,*N*,*O*-tridentate Schiff-base metal complexes in the catalytic ring-opening polymerization of l-lactide, see: Wu *et al.* (2005 ▶); Chen *et al.* (2006 ▶). For a related structure, see: Li *et al.* (2009 ▶).
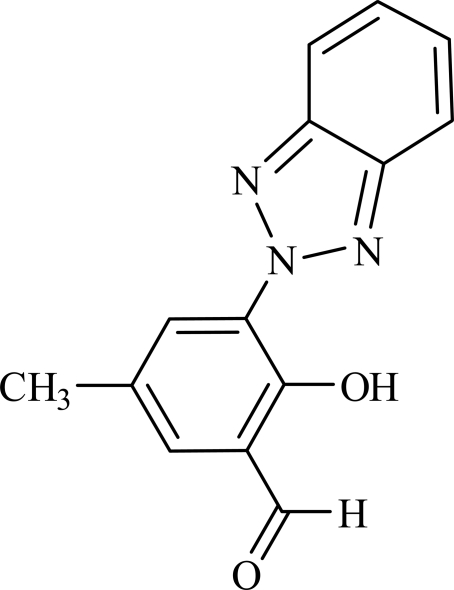


## Experimental

### 

#### Crystal data

C_14_H_11_N_3_O_2_
*M* *_r_* = 253.26
Monoclinic, 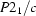

*a* = 12.2724 (5) Å
*b* = 14.5018 (5) Å
*c* = 6.8897 (3) Å
β = 91.571 (2)°
*V* = 1225.71 (8) Å^3^
*Z* = 4
Mo *K*α radiation
μ = 0.10 mm^−1^
*T* = 296 K
0.34 × 0.31 × 0.23 mm

#### Data collection

Bruker APEXII CCD diffractometer
Absorption correction: multi-scan (*SADABS*; Bruker, 2008 ▶) *T*_min_ = 0.972, *T*_max_ = 0.977
13912 measured reflections
2946 independent reflections
1657 reflections with *I* > 2σ(*I*)
*R*_int_ = 0.070

#### Refinement

*R*[*F* ^2^ > 2σ(*F* ^2^)] = 0.045
*wR*(*F* ^2^) = 0.146
*S* = 1.01
2946 reflections
172 parameters
H-atom parameters constrained
Δρ_max_ = 0.21 e Å^−3^
Δρ_min_ = −0.21 e Å^−3^

### 

Data collection: *APEX2* (Bruker, 2008 ▶); cell refinement: *SAINT-Plus* (Bruker, 2008 ▶); data reduction: *SAINT-Plus*; program(s) used to solve structure: *SHELXS97* (Sheldrick, 2008 ▶); program(s) used to refine structure: *SHELXL97* (Sheldrick, 2008 ▶); molecular graphics: *SHELXTL* (Sheldrick, 2008 ▶); software used to prepare material for publication: *SHELXTL*.

## Supplementary Material

Crystal structure: contains datablocks I, global. DOI: 10.1107/S1600536810007233/lh5003sup1.cif

Structure factors: contains datablocks I. DOI: 10.1107/S1600536810007233/lh5003Isup2.hkl

Additional supplementary materials:  crystallographic information; 3D view; checkCIF report

## Figures and Tables

**Table 1:** Hydrogen-bond geometry (Å, °)

*D*—H⋯*A*	*D*—H	H⋯*A*	*D*⋯*A*	*D*—H⋯*A*
O1—H1*A*⋯N1	0.85	1.94	2.588 (2)	132

**Fig. 1.**
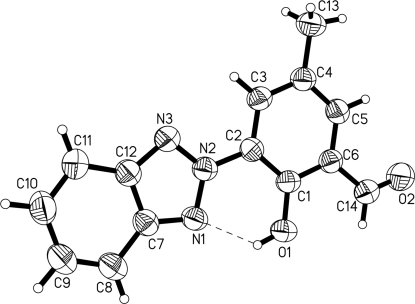
The molecular structure of (I) with the atom numbering scheme. Displacement ellipsoids are drawn at the 50% probability level. The dashed lines indicates a hydrogen bond.

**Fig. 2.**
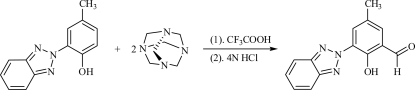
The synthetic procedure of the title compound I.

## supplementary crystallographic information

### Comment

Recently, NNO-tridentate Schiff-base zinc (Zn) and magnesium (Mg) complexes have been attracting considerable attention, mainly due to their applications in the catalytic ring-opening polymerization of L-lactide (Wu *et al.*, 2005; Chen *et al.*, 2006). These NNO-tridentate Schiff-base ligands were easily prepared by condensation from primary amine with the pendant arm of the amino group and various substituted salicylaldehyde derivatives in the presence of MgSO_4_. The additional amino group can be able to provide strong coordination to stabilize Zn or Mg atom and thereby stabilizes the zinc or magnesium alkoxide complex, without further disproportionation. Most recently, our group has successfully synthesized and structural characterized the amino-phenolate ligand derived from 4-methyl-2-(2*H*-benzotriazol-2-yl)-phenol (BTP-H) (Li *et al.*, 2009). In order to develop more useful ligands originated from BTP derivates, our group is interested in the preparation of the multidentate Schiff-base ligand containing the benzotriazol group. Herein, we report the synthesis and crystal structure of the title compound, (I), a potential precursor for the preparation of the multidentate Schiff-base BTP ligands.

The molecular structure of (I) reveals the 5-methylsalicylaldehyde configuration with one benzotriazole functionalized group on the C2-position (Fig. 1). The dihedral angle between the planes of the benzotriazole unit and the benzene ring of the salicylaldehyde group is 2.4 (2)°. There is an intramolecular O—H···N hydrogen bond between the phenol and benzotriazole groups (Table 1). The distance of N1···H1A is substantially shorter than the van der Waals distance of 2.75 Å for the N and H distance. The distances in the benzotriazole-phenolate group are similar to those found in the crystal structure of 2-(2*H*-benzotriazol-2-yl)-6-((diethylamino)methyl)-4-methylphenol (Li *et al.*, 2009).

### Experimental

The title compound (I) was synthesized by the following procedures (Fig. 2): In a 100 ml round bottom flask was placed with 4-methyl-2-(2*H*-benzotriazol-2-yl)phenol (4.50 g, 20.0 mmol) and hexamethylenetetramine (5.60 g, 40 mmol). To this was added trifluoroacetic acid (24 ml, 0.30 mol) and the yellow solution became hot. The resulting mixture was heated to 418 K under reflux for 18 h, during which time the solution colour turned the yellow to dark brown/black. The hot solution was poured into 4 N HCl_(aq)_ (40 ml) and stirred for another 2 h, during which time the solids were formed. The mixture was placed at 253 K overnight and the solids were filtered. The mixture was then extracted with dichloromethane (3 x 150 ml) and the organic layers were dried over MgSO_4_. The final solution was removed the solvent under vacuum to give yellow solids. Yield: 4.40 g (87 %). Single crystals suitable for X-ray diffraction were obtained from a saturated solution of the title compound in Et_2_O.

### Refinement

The H atoms were placed in idealized positions and constrained to ride on their parent atoms, with C–H = 0.93 Å with *U_iso_*(H) = 1.2 *U*_eq_(C) for phenyl hydrogen; 0.96 Å with *U*_iso_(H) = 1.5 *U*_eq_(C) for CH_3_ group; 0.93 Å with *U*_iso_(H) = 1.2 *U*_eq_(C) for CHO group; O–H = 0.85 Å with *U*_iso_(H) = 1.2 *U*_eq_(C).

### Crystal data

**Table:**

C_14_H_11_N_3_O_2_	*F*(000) = 528
*M**_r_* = 253.26	*D*_x_ = 1.372 Mg m^−^^3^
Monoclinic, *P*2_1_/*c*	Mo *K*α radiation, λ = 0.71073 Å
Hall symbol: -P 2ybc	Cell parameters from 1657 reflections
*a* = 12.2724 (5) Å	θ = 1.7–28.3°
*b* = 14.5018 (5) Å	µ = 0.10 mm^−^^1^
*c* = 6.8897 (3) Å	*T* = 296 K
β = 91.571 (2)°	Columnar, yellow
*V* = 1225.71 (8) Å^3^	0.34 × 0.31 × 0.23 mm
*Z* = 4	

### Data collection

**Table:**

Bruker APEXII CCD diffractometer	2946 independent reflections
Radiation source: fine-focus sealed tube	1657 reflections with *I* > 2σ(*I*)
graphite	*R*_int_ = 0.070
Detector resolution: 8.3333 pixels mm^-1^	θ_max_ = 28.3°, θ_min_ = 1.7°
φ and ω scans	*h* = −16→16
Absorption correction: multi-scan (*SADABS*; Bruker, 2008)	*k* = −19→19
*T*_min_ = 0.972, *T*_max_ = 0.977	*l* = −7→9
13912 measured reflections	

### Refinement

**Table:**

Refinement on *F*^2^	Primary atom site location: structure-invariant direct methods
Least-squares matrix: full	Secondary atom site location: difference Fourier map
*R*[*F*^2^ > 2σ(*F*^2^)] = 0.045	Hydrogen site location: inferred from neighbouring sites
*wR*(*F*^2^) = 0.146	H-atom parameters constrained
*S* = 1.01	*w* = 1/[σ^2^(*F*_o_^2^) + (0.075*P*)^2^] where *P* = (*F*_o_^2^ + 2*F*_c_^2^)/3
2946 reflections	(Δ/σ)_max_ < 0.001
172 parameters	Δρ_max_ = 0.21 e Å^−^^3^
0 restraints	Δρ_min_ = −0.20 e Å^−^^3^

### Special details

**Table:**

Experimental. ^1^H NMR (CDCl3, ppm): δ 11.88 (s, 1H, PhOH),10.51 (s, 1H, PhCHO), 8.36 (s, 1H, PhH), 7.94 (d, 2H, PhH), 7.68 (s, 1H, PhH), 7.50 (d, 2H, PhH), 2.41 (s, 3H, PhCH3).
Geometry. All esds (except the esd in the dihedral angle between two l.s. planes) are estimated using the full covariance matrix. The cell esds are taken into account individually in the estimation of esds in distances, angles and torsion angles; correlations between esds in cell parameters are only used when they are defined by crystal symmetry. An approximate (isotropic) treatment of cell esds is used for estimating esds involving l.s. planes.
Refinement. Refinement of *F*^2^ against ALL reflections. The weighted *R*-factor wR and goodness of fit *S* are based on *F*^2^, conventional *R*-factors *R* are based on *F*, with *F* set to zero for negative *F*^2^. The threshold expression of *F*^2^ > σ(*F*^2^) is used only for calculating *R*-factors(gt) etc. and is not relevant to the choice of reflections for refinement. *R*-factors based on *F*^2^ are statistically about twice as large as those based on *F*, and *R*- factors based on ALL data will be even larger.

### Fractional atomic coordinates and isotropic or equivalent isotropic displacement parameters (Å^2^)

**Table:**

	*x*	*y*	*z*	*U*_iso_*/*U*_eq_	
O1	0.50858 (9)	0.36518 (6)	0.26655 (17)	0.0578 (3)	
H1A	0.5768	0.3681	0.2490	0.069*	
O2	0.18809 (10)	0.37151 (8)	0.3322 (2)	0.0816 (4)	
N1	0.68476 (10)	0.27325 (8)	0.20289 (19)	0.0488 (3)	
N2	0.62690 (10)	0.19539 (8)	0.22480 (18)	0.0458 (3)	
N3	0.68234 (10)	0.11695 (8)	0.21302 (19)	0.0517 (4)	
C1	0.45880 (12)	0.28244 (9)	0.2757 (2)	0.0435 (4)	
C2	0.51319 (12)	0.19766 (9)	0.2580 (2)	0.0428 (4)	
C3	0.45656 (12)	0.11543 (10)	0.2707 (2)	0.0471 (4)	
H3B	0.4938	0.0600	0.2579	0.057*	
C4	0.34523 (12)	0.11386 (10)	0.3022 (2)	0.0490 (4)	
C5	0.29195 (12)	0.19740 (10)	0.3180 (2)	0.0493 (4)	
H5A	0.2173	0.1977	0.3381	0.059*	
C6	0.34674 (12)	0.28099 (10)	0.3048 (2)	0.0456 (4)	
C7	0.78649 (11)	0.24258 (10)	0.1756 (2)	0.0476 (4)	
C8	0.88414 (13)	0.29132 (12)	0.1448 (2)	0.0578 (5)	
H8A	0.8858	0.3554	0.1408	0.069*	
C9	0.97489 (13)	0.24055 (13)	0.1215 (2)	0.0637 (5)	
H9A	1.0405	0.2706	0.1011	0.076*	
C10	0.97342 (14)	0.14307 (13)	0.1272 (3)	0.0672 (5)	
H10A	1.0382	0.1111	0.1099	0.081*	
C11	0.88075 (13)	0.09447 (12)	0.1571 (2)	0.0622 (5)	
H11A	0.8806	0.0304	0.1608	0.075*	
C12	0.78537 (12)	0.14584 (11)	0.1820 (2)	0.0491 (4)	
C13	0.28498 (14)	0.02373 (11)	0.3176 (3)	0.0687 (5)	
H13A	0.2093	0.0356	0.3392	0.103*	
H13B	0.3153	−0.0114	0.4241	0.103*	
H13C	0.2919	−0.0105	0.1993	0.103*	
C14	0.28544 (14)	0.36772 (11)	0.3179 (2)	0.0578 (5)	
H14A	0.3241	0.4228	0.3150	0.069*	

### Atomic displacement parameters (Å^2^)

**Table:**

	*U*^11^	*U*^22^	*U*^33^	*U*^12^	*U*^13^	*U*^23^
O1	0.0533 (6)	0.0414 (6)	0.0792 (9)	−0.0065 (5)	0.0092 (5)	−0.0014 (5)
O2	0.0565 (8)	0.0613 (8)	0.1276 (13)	0.0086 (6)	0.0154 (7)	−0.0057 (7)
N1	0.0475 (7)	0.0449 (7)	0.0540 (9)	−0.0062 (6)	0.0031 (6)	0.0029 (6)
N2	0.0458 (7)	0.0421 (7)	0.0495 (9)	−0.0022 (5)	0.0021 (6)	0.0019 (5)
N3	0.0477 (7)	0.0449 (7)	0.0629 (10)	0.0010 (6)	0.0044 (6)	−0.0005 (6)
C1	0.0505 (9)	0.0395 (7)	0.0405 (9)	−0.0044 (6)	0.0012 (7)	−0.0002 (6)
C2	0.0433 (8)	0.0437 (8)	0.0414 (9)	−0.0004 (6)	0.0013 (6)	0.0014 (6)
C3	0.0482 (9)	0.0399 (8)	0.0532 (10)	0.0018 (6)	0.0012 (7)	0.0019 (6)
C4	0.0491 (9)	0.0438 (8)	0.0542 (11)	−0.0018 (6)	0.0017 (7)	0.0028 (7)
C5	0.0444 (8)	0.0508 (9)	0.0530 (10)	−0.0006 (6)	0.0046 (7)	0.0009 (7)
C6	0.0484 (9)	0.0435 (8)	0.0448 (10)	0.0002 (6)	0.0018 (7)	0.0000 (6)
C7	0.0466 (9)	0.0543 (9)	0.0420 (9)	−0.0041 (7)	0.0025 (7)	−0.0001 (7)
C8	0.0539 (10)	0.0615 (10)	0.0583 (12)	−0.0110 (8)	0.0053 (8)	0.0019 (8)
C9	0.0510 (10)	0.0747 (12)	0.0657 (13)	−0.0113 (9)	0.0071 (8)	0.0008 (9)
C10	0.0478 (9)	0.0776 (12)	0.0766 (14)	0.0042 (9)	0.0070 (8)	−0.0052 (9)
C11	0.0516 (10)	0.0583 (10)	0.0771 (13)	0.0046 (8)	0.0076 (8)	−0.0044 (8)
C12	0.0463 (9)	0.0514 (9)	0.0497 (10)	−0.0021 (7)	0.0021 (7)	−0.0006 (7)
C13	0.0560 (10)	0.0495 (10)	0.1008 (15)	−0.0060 (8)	0.0074 (9)	0.0064 (9)
C14	0.0567 (10)	0.0478 (9)	0.0693 (12)	−0.0005 (7)	0.0078 (8)	−0.0033 (7)

### Geometric parameters (Å, °)

**Table:**

O1—C1	1.3488 (15)	C5—H5A	0.9300
O1—H1A	0.8500	C6—C14	1.470 (2)
O2—C14	1.2026 (18)	C7—C12	1.404 (2)
N1—C7	1.3434 (18)	C7—C8	1.412 (2)
N1—N2	1.3445 (16)	C8—C9	1.348 (2)
N2—N3	1.3292 (16)	C8—H8A	0.9300
N2—C2	1.4206 (18)	C9—C10	1.414 (3)
N3—C12	1.3545 (18)	C9—H9A	0.9300
C1—C6	1.395 (2)	C10—C11	1.358 (2)
C1—C2	1.4058 (19)	C10—H10A	0.9300
C2—C3	1.3842 (18)	C11—C12	1.402 (2)
C3—C4	1.389 (2)	C11—H11A	0.9300
C3—H3B	0.9300	C13—H13A	0.9600
C4—C5	1.3823 (19)	C13—H13B	0.9600
C4—C13	1.507 (2)	C13—H13C	0.9600
C5—C6	1.3905 (19)	C14—H14A	0.9300
			
C1—O1—H1A	120.0	C12—C7—C8	120.93 (14)
C7—N1—N2	103.50 (12)	C9—C8—C7	116.85 (16)
N3—N2—N1	116.04 (12)	C9—C8—H8A	121.6
N3—N2—C2	122.44 (11)	C7—C8—H8A	121.6
N1—N2—C2	121.52 (11)	C8—C9—C10	122.10 (15)
N2—N3—C12	103.09 (11)	C8—C9—H9A	119.0
O1—C1—C6	118.00 (13)	C10—C9—H9A	119.0
O1—C1—C2	123.87 (13)	C11—C10—C9	122.29 (16)
C6—C1—C2	118.13 (13)	C11—C10—H10A	118.9
C3—C2—C1	120.50 (14)	C9—C10—H10A	118.9
C3—C2—N2	119.17 (12)	C10—C11—C12	116.62 (16)
C1—C2—N2	120.33 (12)	C10—C11—H11A	121.7
C2—C3—C4	121.44 (13)	C12—C11—H11A	121.7
C2—C3—H3B	119.3	N3—C12—C11	129.87 (15)
C4—C3—H3B	119.3	N3—C12—C7	108.91 (12)
C5—C4—C3	117.86 (13)	C11—C12—C7	121.22 (13)
C5—C4—C13	121.36 (14)	C4—C13—H13A	109.5
C3—C4—C13	120.78 (13)	C4—C13—H13B	109.5
C4—C5—C6	121.88 (14)	H13A—C13—H13B	109.5
C4—C5—H5A	119.1	C4—C13—H13C	109.5
C6—C5—H5A	119.1	H13A—C13—H13C	109.5
C5—C6—C1	120.19 (13)	H13B—C13—H13C	109.5
C5—C6—C14	119.52 (14)	O2—C14—C6	123.75 (15)
C1—C6—C14	120.28 (13)	O2—C14—H14A	118.1
N1—C7—C12	108.46 (12)	C6—C14—H14A	118.1
N1—C7—C8	130.62 (15)		
			
C7—N1—N2—N3	−0.35 (17)	C2—C1—C6—C5	0.8 (2)
C7—N1—N2—C2	179.82 (12)	O1—C1—C6—C14	2.1 (2)
N1—N2—N3—C12	0.42 (17)	C2—C1—C6—C14	−178.04 (13)
C2—N2—N3—C12	−179.75 (12)	N2—N1—C7—C12	0.12 (15)
O1—C1—C2—C3	179.41 (13)	N2—N1—C7—C8	−179.78 (15)
C6—C1—C2—C3	−0.5 (2)	N1—C7—C8—C9	−179.89 (14)
O1—C1—C2—N2	−1.3 (2)	C12—C7—C8—C9	0.2 (2)
C6—C1—C2—N2	178.88 (12)	C7—C8—C9—C10	0.1 (2)
N3—N2—C2—C3	−2.7 (2)	C8—C9—C10—C11	−0.2 (3)
N1—N2—C2—C3	177.08 (13)	C9—C10—C11—C12	0.1 (2)
N3—N2—C2—C1	177.91 (13)	N2—N3—C12—C11	−179.89 (15)
N1—N2—C2—C1	−2.3 (2)	N2—N3—C12—C7	−0.30 (16)
C1—C2—C3—C4	−0.4 (3)	C10—C11—C12—N3	179.71 (16)
N2—C2—C3—C4	−179.71 (13)	C10—C11—C12—C7	0.2 (2)
C2—C3—C4—C5	0.8 (2)	N1—C7—C12—N3	0.12 (16)
C2—C3—C4—C13	−179.41 (15)	C8—C7—C12—N3	−179.97 (14)
C3—C4—C5—C6	−0.5 (2)	N1—C7—C12—C11	179.75 (14)
C13—C4—C5—C6	179.77 (14)	C8—C7—C12—C11	−0.3 (2)
C4—C5—C6—C1	−0.4 (3)	C5—C6—C14—O2	−3.1 (3)
C4—C5—C6—C14	178.51 (14)	C1—C6—C14—O2	175.78 (16)
O1—C1—C6—C5	−179.06 (13)		

### Hydrogen-bond geometry (Å, °)

**Table:**

*D*—H···*A*	*D*—H	H···*A*	*D*···*A*	*D*—H···*A*
O1—H1A···N1	0.85	1.94	2.588 (2)	132
